# Digital Necrosis: A Hoarder’s Tale

**Published:** 2017-09

**Authors:** Arvind Mohan, Justin Conrad Rosen Wormald, Chang Park, Gill Smith

**Affiliations:** **Plastic Surgery Ward, Chelsea and Westminster NHS Foundation Trust, London, UK**

**Keywords:** Hoarding, Self-neglect, Necrosis, Deprivation, Depression

**DEAR EDITOR**

Extreme self-neglect is a problem amongst a small but significant proportion of the population. The hand surgeon encounters cases of acute digital ischemia with a wide spectrum of aetiologies. These include thrombosis, trauma and iatrogenic causes.^1^ A hoarding disorder is characterised by an individual who acquires an excessive number of items and stores them in a chaotic manner.^2 ^Hoarding is a serious concern to communities and to individuals, causing distress to the individual and those around them and putting both at risk of fire, falls, infection and infestation.^2^ The condition is particularly challenging to treat, since individuals have little insight of how it is impacting their lives, with extreme self-neglect being a common problem.^3^^-^^5^ We describe a remarkable case of a patient losing their finger due to completely avoidable circumstances, an extraordinary case of finger necrosis following prolonged external compression from a ring in a chronic hoarder. Indeed, the only reason our patient attended hospital was because his sister made a visit to his home and noticed his swollen, and discoloured finger.

The patient was a 72-year-old unkempt male presented to the emergency department with a grossly necrotic left little finger. The patient described a four-week history of increasing swelling and discolouration affecting the little finger following a minor injury. On examination, the finger was swollen and necrotic secondary to increasing venous congestion from a tight ring. The whole hand was visibly soiled with the presence of faecal matter under the fingernails. 

A fifth ray amputation was performed under general anaesthesia preserving the base of the metacarpal and the extensor carpi ulnaris attachment. The patient attended only one follow up appointment, where it was noted that the wound had almost completely healed. Eventually, social services were contacted, due to safeguarding concerns as a vulnerable adult (Figure 1). 

**Fig. 1:**
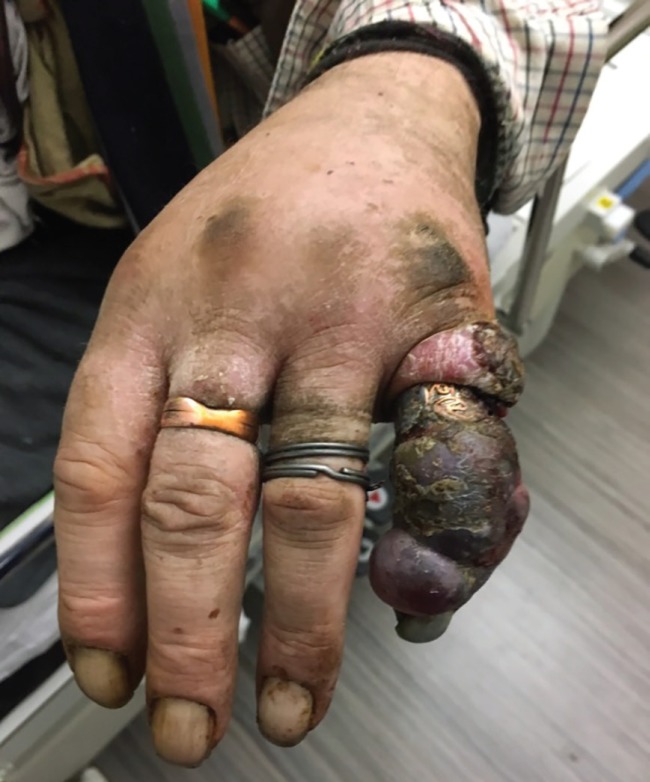
A 72-year-old unkempt male patient with a grossly necrotic left little finger, and increasing swelling and discolouration affecting the little finger following a minor injury

The rings on the middle and ring finger were removed in the emergency department using ring cutters. Following a thorough intra-operative scrub, a fifth ray amputation was performed under general anaesthesia preserving the base of the metacarpal and the extensor carpi ulnaris attachment (Figure 2A and 2B). Post-operatively, the patient was discharged on oral antibiotics and followed up in the dressing clinic, ten days later. He attended his clinic appointment and was reviewed by the plastic surgery specialist nurse. 

**Fig. 2:**
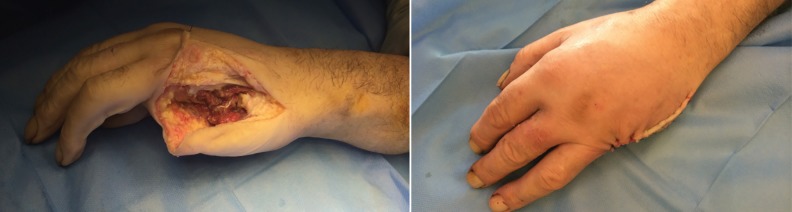
After removal of the rings on the middle and ring finger and a thorough intra-operative scrub, a 5^th^ ray amputation was performed preserving the base of the metacarpal and the extensor carpi ulnaris attachment

He appeared unkempt and the dressing had been soiled. On review of the wound, the proximal aspect had healed well, but there was evidence of small area of wound dehiscence and sloughy discharge more distally. The wound was dressed and he was prescribed further on oral antibiotics. He did not attend any further appointments despite repeated attempts at contacting the patient and eventually social services were contacted, due to safe-guarding concerns as a vulnerable adult.

The pathogenesis of this case was particularly unusual with the reason for progression to such an advanced state likely down to patient factors. It is suspected that the finger became mildly swollen following the initial trauma. The ring was likely to have hampered venous outflow from the digit leading to increasing congestion and edema. Most individuals would, at this stage, have either removed the ring or sought help to remove it. Although removal of rings from digits is a common occurrence for most emergency departments, it is rare that significant arterial ischemia occurs before medical attention is sought. 

In one case, a patient with concurrent psychiatric illness presented with local ischemia of part of the digit, ultimately leading to amputation.^6^ In this case however, the patient presented long before complete ischemia of the digit. In our case report, due to the patient’s social circumstances, psychiatric condition, self-neglect, a vicious cycle of increasing congestion and ischemia led to irreversible tissue necrosis of the entire digit, apparent on presentation to the emergency department. This meant that a different surgical approach was required, as there was no potential for salvage and reconstruction and amputation was indicated immediately. Extreme self-neglect is still a problem amongst a small but significant proportion of the population, so we described a remarkable case of a patient losing their finger due to completely avoidable circumstances. Although removal of rings from digits is a common occurrence for most emergency departments, it is rare that significant arterial ischemia occurs before medical attention is sought.

## CONFLICT OF INTEREST

The authors declare no conflict of interest.

## References

[1] Kadwa AM, Robbs JV (1990). Gangrenous fingers: the tip of the iceberg. Surgeon.

[2] Mataix-Cols D (2014). Clinical practice. Hoarding disorder. N Eng J Med.

[3] Ayers CR, Najmi S, Mayes TL, Dozier ME (2015). Hoarding disorder in older adulthood. Am J Geriatr Psychiatry.

[4] Buscher TP, Dyson J, Cowdell F (2014). The effects of hoarding disorder on families: an integrative review. J Psychiatr Nurs Ment Health Serv.

[5] Ong C, Pang S, Sagayadevan V, Chong SA, Subramaniam M (2015). Functioning and quality of life in hoarding: A systematic review. J Anxiety Disord.

[6] Kumar A, Edwards H, Lidder S, Mestha P (2013). Dangers of neglect: partially embedded ring upon a finger. BMJ Case Rep.

